# Editorial: Cancer metastases: mechanisms of tumor dissemination, formation of metastatic niche and anti-metastatic therapy

**DOI:** 10.3389/fimmu.2025.1746602

**Published:** 2025-11-25

**Authors:** Sergei Kusmartsev, Yingcheng Wu, Elena Voronov, Julia Schueler

**Affiliations:** 1 University of Florida, Gainesville, FL, United States; 2 Zhongshan Hospital, Fudan University, Shanghai, China; 3 Ben-Gurion University of the Negev, Be’er Sheva, Israel; 4 Charles River Laboratories Germany Gesellschaft mit beschränkter Haftung (GmbH), Freiburg, Germany

**Keywords:** cancer metastasis, cancer immuno therapy, immune evasion, tumor dissemination, tumor micro environment (TME), myeloid immunosuppressive cells

Despite decades of progress in treating primary tumors, metastasis remains the final frontier and the central challenge in oncology. At late stages of cancer progression, primary localized tumors may metastasize to distant sites, including bones, lymph nodes, lungs, brain, liver, and other organs. Since current options to treat cancer metastases effectively are highly limited, cancer metastatic disease accounts for approximately 90% of cancer-related deaths. Therefore, there is a critical and unmet need to uncover the mechanisms driving immune resistance and to develop novel, more effective therapeutic strategies. The development of distant tumor metastasis is a highly complex, multi-step process that requires cooperation between cancer and host cells (**Figure 1**), including stromal and immunosuppressive myeloid cells (1–3). While substantial progress has been made in recent years in the biology of tumor metastasis, the precise mechanisms contributing to tumor cell colonization remain elusive, largely because metastasis is not just a cell-autonomous process but an emergent property of a complex host-tumor ecosystem.

**Figure 1:**
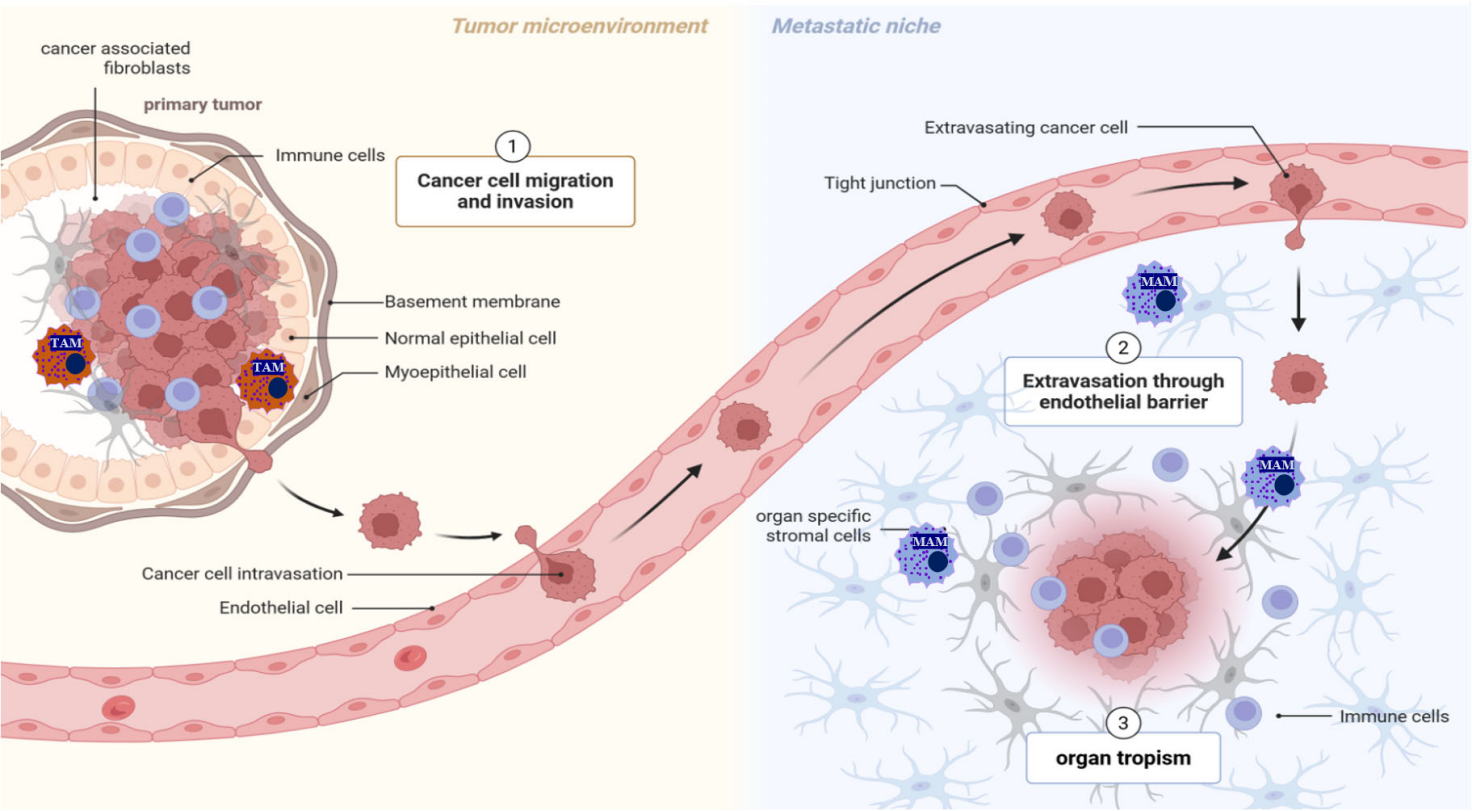
Tumor metastatic colonization to the distant organs is a multi-step process. The development of tumor metastases is not solely determined by the molecular characteristics of cancer cells but also by the intricate interactions between these cells and the surrounding microenvironment, which is composed of specialized cells such as immune cells, activated fibroblasts, endothelial cells, extracellular matrix, and secreted soluble factors.

This Research Topic tackles this multifaceted challenge and aims to provide a comprehensive overview of the molecular, cellular, and organ-level mechanisms of tumor dissemination and the development of novel therapies for the treatment of metastatic disease. Our Research Topic of sixteen manuscripts consists of seven original research papers, eight reviews, and one hypothesis-theory article, which is summarized below. This Research Topic covers different aspects of cancer metastases, including molecular mechanisms of metastatic colonization, roles of stromal, immune cells, and extracellular vesicles in the formation of metastases, mechanisms of immune evasion, and resistance to anti-metastatic therapy. Also, it highlights the efficacy of immunotherapy for the treatment of brain, bone, and liver metastases.

## Anti-metastatic therapy

The recent decade has been marked by significant progress in cancer immunotherapy, particularly in immune checkpoint inhibitors (ICIs) for solid cancers and CAR T cells for hematological malignancies. ICI has revolutionized cancer immunotherapy, yet only a portion of patients with advanced and metastatic cancers experience clinical benefits. Although no clear predictive biomarkers have been developed yet, it is definite that the immunosuppressive tumor microenvironment is a significant barrier to treatment success, as it fosters resistance to immunotherapy.

Wang et al. reviewed the latest research progress in immunotherapy of primary and metastatic bone cancers, including the application status and challenges of major immunotherapy strategies such as ICIs, CAR-T cell therapy, and cancer vaccines. Although osteosarcoma as a primary cancer has a relatively low incidence, it shows high rates of disability and mortality. Metastatic bone cancers frequently arise from many types of solid cancers, including breast, prostate, lung, kidney, bladder, thyroid, and other cancers. Traditional treatments for bone tumors, such as chemotherapy and radiation therapy, have a relatively low efficacy and produce significant side effects. Since immunotherapy has recently shown great potential in improving patient outcomes for many types of cancers, there is hope that this approach will also be effective for the therapy of bone tumors. The results of multiple pre-clinical and clinical studies suggest that, although there are still many challenges, tumor immunotherapy of primary and metastatic bone cancers is expected to make breakthroughs in the near future. This assumption is driven by the in-depth understanding of cellular and molecular mechanisms of bone tumor invasion and immune escape, as well as the development and adoption of novel immunotherapy technologies.

Skalickova et al. provide a detailed overview of immunotherapeutic approaches that focus on the intra-tumoral administration of drugs in patients with locally advanced and metastatic cancers. Intra-tumoral immunotherapy is based on the principle of *in situ* immunization, where the immune response is primed directly within the tumor site, aiming to overcome the immunosuppressive tumor microenvironment and trigger tumor-specific immune responses. The authors describe several types of intra-tumoral therapies, including cancer vaccines (viral oncolytic vaccines, peptide/protein vaccines, and dendritic cell vaccines); intra-tumoral immunomodulators (pattern recognition receptor and stimulator of interferon genes (STING) agonists); intra-tumoral adoptive cell therapies; and intra-tumoral immune checkpoint inhibitors and monoclonal antibodies. Authors acknowledge that most of the intra-tumoral therapies are still in the experimental stage of development, and only T-VEC monotherapy is the only intra-tumoral immunotherapy approved for the treatment of unresectable lesions. Authors conclude that the future of cancer treatment belongs to combinatory therapies that work synergistically to overcome cancer resistance to immunotherapy and enhance the anti-tumor immune response.

In an original preclinical study, Schreiber et al. demonstrated that melanoma tumor growth and its metastatic potential can be inhibited by administration of *Taenia crassiceps* or *Mesocestoides corti* tapeworms in the tumor-bearing mice. Specifically,

Infections with both *T. crassiceps* and *M. corti* significantly enhanced the survival of C57BL/6 mice injected intraperitoneally with B16F10 melanoma cells, compared to mice that received only melanoma. While only 2 out of 10 mice in the melanoma-only group survived after 26 days, all mice infected with *M. corti*, and 9 out of 10 infected with T*. crassiceps*, survived until the end of the observation period. However, the authors acknowledge that although helminth parasites may have a potential role in combating cancer, it is important to recognize that they are pathogenic organisms capable of causing harm to the host when introduced as whole parasites. Consequently, further research is necessary to elucidate the local mechanisms, both immunological and non-immunological, that contribute to the protective effect of tapeworm infection against cancer. The review by Xu et al. focused on survival outcomes and risk profiles of patients with thyroid cancers with distant liver metastases. The clinico-pathological characteristics of patients with and without liver metastases were compared. Additionally, they evaluated the prognostic outcomes of these patients in the context of the immunotherapy era. The prevalence of liver metastasis in thyroid cancer was 0.22% (95% CI 0.20%-0.25%), mainly occurring in medullary and anaplastic thyroid carcinomas. However, there was no statistically significant difference in median overall survival or median cancer-specific survival between the pre- and post-immunotherapy periods.

## Roles of immunosuppression and immune evasion in metastatic spreading

A critical issue in tumor metastasis is the survival of cancer cells upon the invasion of a distant organ. To survive and grow in newly invaded tissue, metastatic tumor cells interact with the local stromal cells and extracellular matrix to obtain vital survival signals. Besides, the metastatic cancer cells must evade detection and destruction by the host’s immune system. To escape from immune response, cancer cells develop a tolerogenic microenvironment through the secretion of immunosuppressive factors and attracting bone marrow-derived myeloid cells, which inhibit the anti-tumor immune response mediated by T lymphocytes and NK cells (3–5). In addition, cancer cells can reduce immunogenicity and evade the immune surveillance by downregulation of MHC class I on the surface of cancer cells.

The work of Arias-Badia et al. provides evidence that exposure to E-cigarettes disrupts the anti-tumor immunity and promotes metastases. The authors investigated the effects of the two main components of e-cigarettes, PG/VG and nicotine, on tumor development using preclinical animal models. Their findings showed that PG/VG enhances tumor cell motility, while nicotine reduces tumor cell proliferation *in vitro*. Additionally, both tumor-infiltrating and circulating T cells in e-cigarette-exposed mice showed increased levels of immune checkpoint markers, such as CTLA4 and PD-1. The authors conclude that the key constituents of e-cigarette fluid can influence tumor progression by inducing immunosuppression, leading to the accumulation of functionally exhausted T cells expressing PD-1 and CTLA4. The original study by Bauer et al. describes how human myeloid sarcomas (MS) escape the immune system response. This disease is characterized by rare extramedullary manifestations of myeloid neoplasms, often associated with poor patient prognosis. Through immunohistochemistry, multispectral imaging, and RNA sequencing of bone marrow samples from MS patients, the authors determined that reduced HLA-I expression and a low density of tumor-infiltrating lymphocytes (TILs) were associated with worse clinical outcomes. Ma et al. summarize the roles of altered lipid metabolism in ovarian cancer in shaping an immunosuppressive tumor microenvironment that promotes tumor growth and development of metastases. Authors describe the alteration of lipid uptake, *de novo* fatty acid synthesis, lipid oxidation, and lipid storage in ovarian cancer. As noted in this review, the metabolic dysfunction exists not only in tumor cells but is widely detected in stromal cells and tumor-infiltrating immune cells. In a lipid-rich tumor microenvironment, functional changes primarily affect stromal and immune cells, such as CAFs, Tregs, CD8 T cells, and tumor-associated macrophages. Additionally, interactions among these cells create a premetastatic niche and an immunosuppressive tumor microenvironment, facilitating metastasis and tumor immune evasion, which ultimately enhances the proliferative characteristics of ovarian cancer.

## Molecular mechanisms of metastasis development

Establishing the biological mechanisms of the metastatic process is crucial for understanding the precise molecular and cellular events contributing to tumor cell colonization and the development of new anti-metastatic therapies. Feigelman et al. demonstrated that EMMPRIN (CD147) promotes spheroid tumor cell organization and metastatic formation. EMMPRIN is known as a hub protein that stabilizes large protein complexes, such as CD44, MCT1/4, CD99, and integrins, and is implicated in the regulation of tumor cell proliferation. Data obtained by the authors indicate that knockdown of EMMPRIN expression in murine CT26 tumor cells resulted in inhibited cell proliferation and reduced angiogenic potential, while enhancing tumor resistance to drugs. In addition, the absence of EMMPRIN prevented tumor cells from forming metastatic-like lesions when seeded on basement membrane extract. CD44 (cluster of differentiation 44) is a multifunctional transmembrane glycoprotein involved in cell-cell interaction, adhesion, and cell migration. CD44 has previously been reported as stem cell marker and key driver in cancer progression and metastasis development. The study by Maltseva et al. shows that deletion of the CD44 gene in HT-29 colorectal cancer cells significantly alters the expression of several miRNAs and their target genes. The data obtained suggest that CD44 is closely involved in the regulatory relationship between Let-7 miRNA and STAT3 in cancer cells.

Albakova summarizes the functions of 90-kDa heat shock proteins (HSP90) in primary and metastatic cancers. HSP90 chaperones interact with a wide and diverse array of proteins, many of which play critical roles in tumor development, immune evasion, invasion, and metastasis. Due to its significant role in cancer, HSP90 has emerged as a promising target for anti-cancer therapies. Several clinical trials have explored an HSP90 inhibitor combined with verteporfin, a photosensitizer, and a near-infrared red probe for diagnostic imaging of solid tumors. Leveraging HSP90’s ability to stimulate anti-tumor responses, various HSP90-based immunotherapies have been developed. Nevertheless, no HSP90 inhibitors have received FDA approval to date. The author suggests that further understanding of the complex functions of HSP90 in cancer could open new avenues for diagnosis and treatment of cancer patients. Wang et al. reviewed recent progress on the roles of miRNA-136 in the cancer metastatic process. miR-136 is aberrantly expressed in numerous metastatic tumors and is closely linked to tumor cell proliferation, apoptosis, invasion, and metastasis, highlighting its significant role in tumor growth and progression. MicroRNAs (miRNAs) modulate the expression of their target genes and can exert either carcinogenic or tumor-suppressive effects, directly or indirectly, across different types of cancer. The miR-136 regulates various molecular signaling pathways, including Wnt, MAP2K4-JNK, PTEN, MTDH, LRH1, and others. The authors suggest that broad roles in the regulation of tumor growth make miR-136 a potential therapeutic target. In the original study, Lin et al. showed that the circular RNA Circ-0007552 inhibits the progression of lung cancer metastases in a preclinical model. Specifically, both *in vitro* and *in vivo* experiments revealed that overexpression of Circ-0007552 reduced the malignant behaviors of lung cancer cells, while its knockdown produced opposite effects. Mechanistically, Circ-0007552 acts as a competing endogenous RNA (ceRNA) for miR-7974, suppressing its expression.

## Extracellular vesicles in cancer metastases

Exosomes or extracellular vesicles (EVs) in cancer are tiny particles released by tumor cells that act as mediators of cell-to-cell communication, playing a significant role in cancer progression, metastasis, and immune evasion. They are involved in a wide range of cancer-related processes, including tumor growth, angiogenesis (blood vessel formation), immune regulation, and the formation of pre-metastatic niches in distant organs. Chen et al. provides a review on the roles of EVs in facilitating lung cancer metastasis and their influence on disease progression and spread to distant tissues. It also explores the potential of exosomes as biomarkers for lung cancer metastasis, providing valuable insights for future clinical applications. Highlighting the various mechanisms through which exosomes promote lung cancer dissemination to the brain and bone, the review suggests potential targets for intervention in the diagnosis and treatment of metastatic lung cancer. Nidhi et al. provide an analysis of molecular mechanisms of organotropic metastatic colonization orchestrated by EVs. Focusing on immune modulation and tumor microenvironment reprogramming, the authors present a comprehensive review of EV biogenesis and cargo composition. They explore the connection between EV types and their molecular cargo, along with the regulatory mechanisms governing EV formation and release. The review highlights EV functions in intercellular communication and their critical roles in establishing pre-metastatic niches. Furthermore, EVs are examined as potential diagnostic and prognostic biomarkers for metastasis. The authors acknowledge current limitations and challenges in EV research and clinical application, such as the lack of standardized protocols for EV isolation, characterization, and quantification, which hinder reproducibility and clinical translation efforts.

## The impact of exercise on metastatic development

The original preclinical study of Stagaard et al. provides evidence that a boost of physical activity following surgery may be beneficial in delaying breast cancer metastatic development. In this study, the authors used mice with implanted highly metastatic 4T1 tumor cells for the modeling of breast metastatic cancer. Thus, voluntary wheel running was found to notably enhance metastasis-free survival, effectively doubling the median survival time. However, these benefits were only seen when an increase in physical activity occurred after surgery. To explore this further, the authors conducted mock surgeries and verified that surgical stress was essential for the beneficial impact of increased exercise on lowering metastatic tumor burden in mice with either spontaneous or experimentally induced metastasis. The authors conclude that exercise and a boost of physical activity following surgery result in a delay of metastatic development.

## Conclusion

Overall, the knowledge offered in these articles is beneficial for building a clearer picture of cancer metastasis biology. However, much more extensive investigations on this Research Topic are still required for improving our understanding of the mechanisms of metastatic colonization, to develop novel therapeutic approaches, and boost the efficiency of existing anti-metastatic therapy. We must move beyond targeting only the seed (the cancer cell) and increasingly focus on reprogramming the soil (the host micro- and macro-environment). This necessitates the development of novel therapeutic combinations (e.g., combining ICIs with metabolic modulators or STING agonists) and, crucially, preclinical models that can better recapitulate these complex, systemic host-tumor interactions.
